# A Time-Delayed Mathematical Model for Tumor Growth with the Effect of a Periodic Therapy

**DOI:** 10.1155/2016/3643019

**Published:** 2016-05-05

**Authors:** Shihe Xu, Xiangqing Wei, Fangwei Zhang

**Affiliations:** ^1^School of Mathematics and Statistics, Zhaoqing University, Zhaoqing, Guangdong 526061, China; ^2^Teaching Research Administration of Guangrao County, Dongying, Shandong 257300, China; ^3^College of Transport and Communications, Shanghai Maritime University, Shanghai 201306, China

## Abstract

A time-delayed mathematical model for tumor growth with the effect of periodic therapy is studied. The establishment of the model is based on the reaction-diffusion dynamics and mass conservation law and is considered with a time delay in cell proliferation process. Sufficient conditions for the global stability of tumor free equilibrium are given. We also prove that if external concentration of nutrients is large the tumor will not disappear and the conditions under which there exist periodic solutions to the model are also determined. Results are illustrated by computer simulations.

## 1. Introduction

The process of tumor growth is one of the most intensively studied processes in recent years. There have appeared many papers devoted to develop mathematical models to describe the process (see, e.g., [1–8]). Most of those models are based on the reaction-diffusion equations and mass conservation law. The process of tumor growth has several different stages, starting from the very early stage of solid tumor without necrotic core inside (see, e.g., [2, 9–12]) to the process of necrotic core formation (see, e.g., [3, 13–15]). Experiments suggest that changes in the proliferation rate can trigger changes in apoptotic cell loss and that these changes do not occur instantaneously: they are mediated by growth factors expressed by the tumor cells (see [13]). Following this idea, the study of time-delayed mathematical model for tumor growth has drawn attention of some other researchers (see, e.g., [6, 11, 16–18] and references cited therein).

At the beginning, we formulate the model. In the model we assume that the tumor is nonnecrotic and consider two unknown functions:

(i) *σ*(*r*, *t*): the nutrient concentration at radius *r* and time *t*,
(ii) *R*(*t*): the outer tumor radius at time *t*.

It is assumed that the consumption rate of nutrient is proportional to the local nutrient concentration. Denoting by Γ the coefficient of proportionality, then the changes of *σ* are described by the following reaction-diffusion equation:

(1) $$\begin{array}{c} \frac{1}{r^{2}}\frac{\partial }{\partial r}\left(\;r^{2}\frac{\partial \sigma }{\partial r}\;\right)=\Gamma \sigma ,\;0<r<R\left(t\right),\;\;t>0. \end{array}$$

The changes of *R* are governed by the mass conservation law, that is,

(2) $$\begin{array}{c} \frac{d}{dt}\left(\;\frac{4\pi R^{3}}{3}\;\right)=S-Q-P, \end{array}$$

where *S*, *Q*, and *P* denote the net rates of proliferation, natural apoptosis, and apoptosis caused by therapy, respectively. It is reasonable to assume that the proliferation rate is proportional to the local nutrient concentration. Denoting the coefficient of proportionality by *s*, we obtain

(3) $$\begin{array}{c} S=4\pi \overset{R\left(t-\tau \right)}{\underset{0}{\int }}s\sigma \left(\;r,t-\tau \;\right)r^{2}dr, \end{array}$$

where we denote by *τ* the time delay in cell proliferation; that is, *τ* is the length of the period that a tumor cell undergoes a full process of mitosis. It is assumed that the apoptotic cell loss occurs with a constant rate $s\tilde{\sigma }$, that is,

(4) $$\begin{array}{c} Q=4\pi \overset{R\left(t\right)}{\underset{0}{\int }}s\tilde{\sigma }r^{2}dr. \end{array}$$

It is assumed that the cell apoptosis caused by the periodic therapy occurs with a periodic rate *sλ*(*t*), that is,

(5) $$\begin{array}{c} P=4\pi \overset{R\left(t\right)}{\underset{0}{\int }}s\lambda \left(\;t\;\right)r^{2}dr, \end{array}$$

where *λ*(*t*) is a positive periodic function with period *ω*. The boundary conditions are as follows:

(6) ∂σ∂r0,t=0,σRt,t=σ∞,0<r<Rt,  t>0,

where the constant *σ*_*∞*_ denotes the external concentration of nutrients.

We will consider (1)-(2) together with the following initial condition:

(7) $$\begin{array}{c} R\left(\;t\;\right)=\varphi \left(\;t\;\right),\;-\tau \leq t\leq 0. \end{array}$$

The idea of considering the effect of periodic therapy is motivated by [17]. In [17], through experiments, the authors observed that after an initial exponential growth phase leading to tumor expansion, growth saturation is observed even in the presence of periodically external condition. In this paper, we mainly discuss how the periodic therapy affects the growth of the avascular tumor. The model studied in this paper is similar to the first model studied in [11] and the model discussed in [19], but with some modifications. In [11, 19], the authors only consider the special cases of the model. In [11], the authors consider the case where *λ*(*t*) ≡ 0 and in [19], the author considers the case where *τ* = 0. In this paper, we will consider the general model in which *τ* > 0 and *λ*(*t*) is a periodic function. It should be pointed out that the methods used in [11, 19] are no longer applicable. In this paper, by the fixed point index theorem, the conditions under which there exist periodic solutions to the model are determined. Using the comparison principle, sufficient conditions for the global stability of tumor free equilibrium are given. Results are illustrated by computer simulations.

## 2. Analytical Results

By rescaling the space variable we may assume that Γ = 1. Accordingly, the solution to (1), (6) is

(8) $$\begin{array}{c} \sigma \left(\;r,t\;\right)=\frac{\sigma_{\infty }R\left(t\right)}{\sinh R\left(t\right)}\frac{\sinh r}{r}. \end{array}$$

Substituting (8) to (2), one can get

(9) 1sdRdt=Rtσ∞pRt−τRt−τRt3−σ~3−λt3,

where

(10) $$\begin{array}{c} p\left(\;x\;\right)=\frac{x\coth x-1}{x^{2}}. \end{array}$$

Denote *x* = *R* ^3^ and assume that *s* = 1 (if not one can rescale coefficients *σ*_*∞*_, $\tilde{\sigma }$, and *λ*(*t*)). Then (9) takes the form

(11) $$\begin{array}{c} \frac{dx}{dt}=3\sigma_{\infty }f\left(\;x\left(\;t-\tau \;\right)\;\right)-\gamma \left(\;t\;\right)x\left(\;t\;\right), \end{array}$$

where $f(x)=xp(\sqrt[{3}]{x})$, $\gamma (t)=\tilde{\sigma }+\lambda (t)$. Accordingly, the initial condition takes the following form:

(12) $$\begin{array}{c} x_{0}\left(\;t\;\right)=\varphi^{3}\left(\;t\;\right),\;-\tau \leq t\leq 0. \end{array}$$

By the method of steps it is clear that the initial value problem (11), (12) has a unique solution *x*(*t*) which exists for all *t* ≥ 0, because we may rewrite this problem in the following functional form:

(13) xt=x00e−∫0tγsds+3σ∞e−∫0tγsds∫0te∫0ξγsdsfxξ−τdξ.

Since *f*(*x*) ≥ 0 for all *x* ≥ 0, then, by Theorem  1.1 [20], we have the solution of problem (11), (12) being nonnegative on the interval on which it exists.

In order to prove our results, we should use the following Lemma from [11].

**Lemma 1 (see [11]).**

Consider the initial value problem of a delay differential equation:

(14) x˙t=gxt,xt−τfor  t>0xt=x0tfor  −τ≤t≤0.

Assume that the function *g* is defined and continuously differentiable in *R*_+_ × *R*_+_ and strictly monotone increasing in the second variable; we have the following results:

(1) If *x*_*s*_ is a positive solution of equation *g*(*x*, *x*) = 0 such that *g*(*x*, *x*) > 0 for *x* less than but near *x*_*s*_, *g*(*x*, *x*) < 0 for *x* greater than but near *x*_*s*_. Let (*c*, *d*) be the maximal interval containing only the root *x*_*s*_ of equation *g*(*x*, *x*) = 0. If *x*(*t*) is the solution of the problem of (14) and *x*_0_(*t*) ∈ *C*[−*τ*, 0], *c* < *x* ^0^(*t*) < *d* for −*τ* ≤ *t* ≤ 0. Then
  (15) $$\begin{array}{c} \underset{t\to \infty }{\lim}x\left(\;t\;\right)=x_{s}. \end{array}$$
(2) Assume further that *g*(*x*, *x*) is negative for small *x* > 0, and let *b* be the first positive root of the equation *g*(*x*, *x*) = 0 (if *g*(*x*, *x*) < 0 for all *x* > 0, then we define *b* = *∞*). If *x*_0_(*t*)∈(0, *b*) for all −*τ* ≤ *t* ≤ 0 and the solution to (14) exists for all *t* ≥ −*τ*, then
  (16) $$\begin{array}{c} \underset{t\to \infty }{\lim}x\left(\;t\;\right)=0. \end{array}$$

**Lemma 2 .**

(1) *p*(*x*) is monotone decreasing for all *x* > 0 and

(17) limx→0+⁡px=13,limx→+∞⁡px=0.

(2) *x* ^3^ *p*(*x*) is monotone increasing for all *x* > 0.

**Proof**

For (1) please see [12] and for (2) see [11]. This completes the proof.

In the following, we assume that *λ*(*t*) is a continuous function on *R*. Denote

(18) λ−=1ω∫0ωλtdt,λ∗=max0≤t≤ω⁡λt,λ∗=min0≤t≤ω⁡λt>0

and assume that *s* = 1 (if not one can rescale coefficients *σ*_*∞*_, $\tilde{\sigma }$, and *λ*(*t*)).

By (11) and Lemma 2(1), we have

(19) $$\begin{array}{c} \frac{dx}{dt}\leq \left[\;\sigma_{\infty }-\gamma \left(\;t\;\right)\;\right]x\left(\;t\;\right). \end{array}$$

It follows that when $\sigma_{\infty }<\tilde{\sigma }$, cancer will be eliminated even without therapy. This makes the analysis of the model with therapy worthwhile only in the case where $\sigma_{\infty }>\tilde{\sigma }$. Here and hereafter, we assume that the condition $\sigma_{\infty }>\tilde{\sigma }$ holds.

**Lemma 3 .**

If *λ* ≡ *λ*_0_, where *λ*_0_ is a positive constant, the following assertions hold:

(1) If $\lambda_{0}<\sigma_{\infty }-\tilde{\sigma }$, (11) has a unique positive stationary point *x*_*s*_ which is determined by $p(\sqrt[{3}]{x})=\left(\tilde{\sigma }+\lambda_{0}\right)/3\sigma_{\infty }.$ If $\lambda_{0}\geq \sigma_{\infty }-\tilde{\sigma }$, (11) has no positive stationary solution.
(2) If $\lambda_{0}<\sigma_{\infty }-\tilde{\sigma }$, all solutions of (11) which are positive in the initial interval [−*τ*, 0] exist for all *t* ≥ −*τ* and converge to *x*_*s*_ as *t* → *∞*. If $\lambda_{0}\geq \sigma_{\infty }-\tilde{\sigma }$, then all solutions of (11) which are positive in [−*τ*, 0] also exist for all *t* ≥ −*τ* and they converge to zero as *t* → *∞*.

**Proof**

(1) If *λ* ≡ *λ*_0_, where *λ*_0_ is a positive constant, that is, $\overset{-}{\lambda }=\lambda^{*}=\lambda_{*}=\lambda_{0}$, then the stationary solutions of (11) satisfy the following equation:

(20) $$\begin{array}{c} 3\sigma_{\infty }f\left(\;x\;\right)-\left[\;\tilde{\sigma }+\lambda_{0}\;\right]x=0; \end{array}$$

that is,

(21) $$\begin{array}{c} \left[\;3\sigma_{\infty }p\left(\;\sqrt[{3}]{x}\;\right)-\left(\;\tilde{\sigma }+\lambda_{0}\;\right)\;\right]x=0. \end{array}$$

By Lemma 2(1), one can get the following assertions: if $\lambda_{0}<\sigma_{\infty }-\tilde{\sigma }$, (11) has a unique positive stationary point *x*_*s*_ which is determined by $p(\sqrt[{3}]{x})=\left(\tilde{\sigma }+\lambda_{0}\right)/3\sigma_{\infty }$. If $\lambda_{0}\geq \sigma_{\infty }-\tilde{\sigma }$, (11) has no positive stationary solution.

(2) Set $g(x(t),x(t-\tau ))=3\sigma_{\infty }f(x(t-\tau ))-(\tilde{\sigma }+\lambda_{0})x(t)$; then $g(x,y)=3\sigma_{\infty }f(y)-(\tilde{\sigma }+\lambda_{0})x$. By simple computation

(22) $$\begin{array}{c} \frac{\partial g}{\partial y}=3\sigma_{\infty }f^{\prime }\left(\;y\;\right)=\sigma_{\infty }\left[\;3p\left(\;\sqrt[{3}]{y}\;\right)+\sqrt[{3}]{y}p^{\prime }\left(\;\sqrt[{3}]{y}\;\right)\;\right]. \end{array}$$

From Lemma 2(2), we know

(23) $$\begin{array}{c} {\left(\;x^{3}p\left(\;x\;\right)\;\right)}^{\prime }=x^{2}\left[\;p\left(\;x\;\right)+xp^{\prime }\left(\;x\;\right)\;\right]>0 \end{array}$$

for all *x* > 0; it follows that *p*(*x*) + *xp*′(*x*) > 0 for all *x* > 0. Thus, $3p(\sqrt[{3}]{y})+\sqrt[{3}]{y}p^{\prime }(\sqrt[{3}]{y})>0$ for all *y* > 0. Therefore, *g* is strictly monotone increasing in the second variable.

By monotonicity of the function *p* and Lemma 3(1), we can get the following: if $\lambda_{0}<\sigma_{\infty }-\tilde{\sigma }$, then *g*(*x*, *x*) > 0 for *x* < *x*_*s*_; *g*(*x*, *x*) < 0 for *x* > *x*_*s*_. By Lemma 1(1), for any nonnegative initial function *x*_0_(*t*), the following holds:

(24) $$\begin{array}{c} \underset{t\to \infty }{\lim}x\left(\;t\;\right)=x_{s}. \end{array}$$

If $\lambda_{0}\geq \sigma_{\infty }-\tilde{\sigma }$, we have $g(x,x)=x[3\sigma_{\infty }p(\sqrt[{3}]{x})-(\tilde{\sigma }+\lambda_{0})]<0$ for all *x* > 0. Then lim_*t*→*∞*_ *x*(*t*) = 0 follows from Lemma 1(2). This completes the proof of Lemma 3.

**Figure 1:**
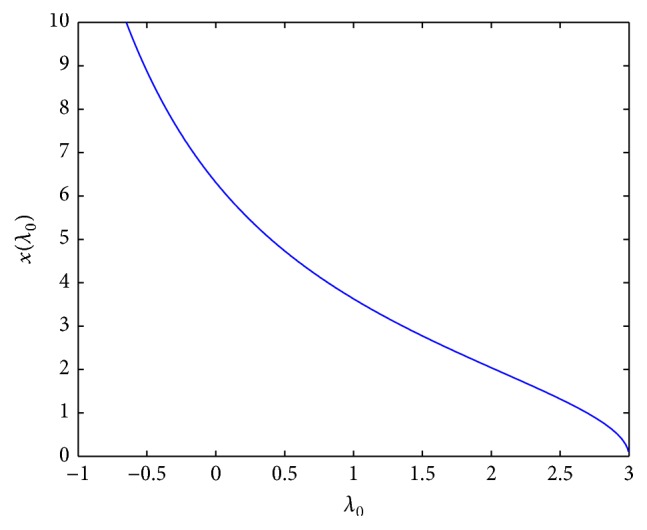
The diagram of $x_{s}(\lambda_{0})={\left[p^{-1}(\left(\tilde{\sigma }+\lambda_{0}\right)/3\sigma_{\infty })\right]}^{3}$, where *σ*_*∞*_ = 5, and $\tilde{\sigma }=2$.

In Figure 1, an example of the graph of *x*_*s*_(*λ*_0_) is presented which is covered by Lemma 3(1), where *σ*_*∞*_ = 5 and $\tilde{\sigma }=2$.

Consider the following two equations:

(25) f1x3σ∞fx−γ∗x=3σ∞px3−γ∗x=0,f2x3σ∞fx−γ∗x=3σ∞px3−γ∗x=0,

where $\gamma^{*}=\tilde{\sigma }+\lambda^{*}$, $\gamma_{*}=\tilde{\sigma }+\lambda_{*}$. If $\lambda^{*}<\sigma_{\infty }-\tilde{\sigma }$, one can get

(26) $$\begin{array}{c} 0<\frac{\gamma_{*}}{3\sigma_{\infty }}\leq \frac{\gamma^{*}}{3\sigma_{\infty }}<\frac{1}{3}. \end{array}$$

By Lemma 1(1), we know that the function *p* is monotone decreasing and 0 < *p*(*y*) < 1/3 for any *y* > 0. Therefore, the above two equations have a unique positive constant solutions *x*_1_ and *x*_2_, respectively; that is, there exists a unique constant *x*_1_ > 0 such that *f*_1_(*x*_1_) = 0 and a unique constant *x*_2_ > 0 such that *f*_2_(*x*_2_) = 0. By the fact that *p*(*y*) is monotone decreasing for any *y* > 0, we can get *x*_2_ > *x*_1_ > 0.

**Theorem 4 .**

(i) If $\lambda^{*}<\sigma_{\infty }-\tilde{\sigma }$, then, for any nonnegative initial function *x*(*t*), there exists *T* > 0 such that the unique solution *x*(*t*) to (11), (12) satisfies *x*(*t*)∈[*x*_1_/2, 3*x*_2_/2] for *t* ≥ *T*.

(ii) If $\lambda_{*}\geq \sigma_{\infty }-\tilde{\sigma }$, then, for any nonnegative initial function *x*(*t*), the unique solution *x*(*t*) to (11), (12) satisfies lim_*t*→*∞*_ *x*(*t*) = 0.

**Proof**

(i) By (11), one can get

(27) 3σ∞fxt−τ−σ~+λ∗xt≤dxdt≤3σ∞fxt−τ−σ~+λ∗xt.

Consider the following initial value problem:

(28) dxdt=gxt,xt−τ,x0t=φ3t,−τ≤t≤0,

where $g(x,y)=3\sigma_{\infty }f(y)-(\tilde{\sigma }+\lambda_{*})x$. With similar arguments as that in Lemma 3(2), one can get *g* strictly monotone increasing in the second variable.

Since *g*(*x*, *x*) = *f*_2_(*x*) and

(29) $$\begin{array}{c} 0<\frac{\gamma_{*}}{3\sigma_{\infty }}\leq \frac{\gamma^{*}}{3\sigma_{\infty }}<\frac{1}{3}, \end{array}$$

where $f_{2}(x)=3\sigma_{\infty }f(x)-\gamma_{*}x=[3\sigma_{\infty }p(\sqrt[{3}]{x})-\gamma_{*}]x$ as before, $\gamma^{*}=\tilde{\sigma }+\lambda^{*}$, $\gamma_{*}=\tilde{\sigma }+\lambda_{*}$. By the fact that the function *p*(*x*) is monotone decreasing and 0 < *p*(*y*) < 1/3 for any *y* > 0, one can get *g*(*x*, *x*) = *f*_2_(*x*) = 0 having a unique positive constant solution *x*_*s*_ = *x*_2_, and *g*(*x*, *x*) > 0 for *x* < *x*_2_; *g*(*x*, *x*) < 0 for *x* > *x*_2_. By Lemma 1, we have, for any nonnegative initial function *x*_0_(*t*),

(30) $$\begin{array}{c} \underset{t\to \infty }{\lim}x\left(\;t\;\right)=x_{2}. \end{array}$$

Similarly by considering the following initial value problem,

(31) dxdt=3σ∞fxt−τ−σ~+λ∗xt,x0t=φ3t,−τ≤t≤0,

one can get, for any nonnegative initial function *x*_0_(*t*),

(32) $$\begin{array}{c} \underset{t\to \infty }{\lim}x\left(\;t\;\right)=x_{1}. \end{array}$$

By (30) and (32) and a comparison principle (cf. Lemma  3.1 in [11]), we can get that there exists *T* > 0 such that the unique solution *x*(*t*) to (11), (12) satisfies that *x*(*t*)∈[*x*_1_/2, 3*x*_2_/2] for *t* ≥ *T*.

(ii) The proof is similar to that of Lemma 3(2) by considering (28); we omit it here. This completes the proof of Theorem 4.

**Remark 5 .**

Note that *x*_1_ and *x*_2_ are decreasing function of *λ* ^*∗*^ and *λ*_*∗*_, respectively; one can easily get that when *λ* ^*∗*^ − *λ*_*∗*_ decreases, 3*x*_2_/2 − *x*_1_/2 will decrease; that is, the interval [*x*_1_/2, 3*x*_2_/2] will be reduced.

In the following, we will give some results that (11) admits an oscillatory solution whose period matches that of *λ*(*t*).

Let

(33) $$\begin{array}{c} X=\left\{\;x:x\in C\left(\;R,R\;\right),x\left(\;t+\omega \;\right)=x\left(\;t\;\right)\;\right\}, \end{array}$$

with the usual linear structure as well as the norm

(34) $$\begin{array}{c} \left\|\;x\;\right\|=\underset{t\in \left[0,\omega \right]}{sup}\left|\;x\left(\;t\;\right)\;\right|. \end{array}$$

Then *X* is a Banach space. Define

(35) $$\begin{array}{c} K=\left\{\;x\in X:x\left(\;t\;\right)\geq \kappa \left\|\;x\;\right\|,\;\;t\in \left[\;0,\omega \;\right]\;\right\}, \end{array}$$

where $\kappa =e^{-\int_{0}^{\omega }(\tilde{\sigma }+\lambda (t))dt}=e^{-\omega (\tilde{\sigma }+\overset{-}{\lambda })}$. Then *K* is a cone in *X*. By (11), we have

(36) $$\begin{array}{c} x\left(\;t\;\right)=3\sigma_{\infty }\overset{t+\omega }{\underset{t}{\int }}G\left(\;t,s\;\right)f\left(\;x\left(\;s-\tau \;\right)\;\right)ds, \end{array}$$

where $G(t,s)=e^{\int_{t}^{s}\gamma (u)du}/\left(e^{\int_{0}^{\omega }\gamma (u)du}-1\right)=e^{\int_{t}^{s}\gamma (u)du}/\left(e^{(\tilde{\sigma }+\overset{-}{\lambda })\omega }-1\right)=e^{\int_{t}^{s}\gamma (u)du}/\left(\kappa^{-1}-1\right)$, *s* ∈ [*t*, *t* + *ω*].

It is easy to see that (11) admits oscillatory solutions whose period matches that of *λ*(*t*) if and only if (36) has *ω*-periodic solutions. Further, one can get

(37) $$\begin{array}{c} \frac{1}{\kappa^{-1}-1}\leq G\left(\;t,s\;\right)\leq \frac{\kappa^{-1}}{\kappa^{-1}-1},\;s\in \left[t,t+\omega \right]; \end{array}$$

that is,

(38) $$\begin{array}{c} \frac{\kappa }{1-\kappa }\leq G\left(\;t,s\;\right)\leq \frac{1}{1-\kappa },\;s\in \left[t,t+\omega \right]. \end{array}$$

Define an operator *A* : *X* → *X* by

(39) $$\begin{array}{c} \left(\;Ax\;\right)\left(\;t\;\right)=3\sigma_{\infty }\overset{t+\omega }{\underset{t}{\int }}G\left(\;t,s\;\right)f\left(\;x\left(\;s-\tau \;\right)\;\right)ds. \end{array}$$

Then the following assertions hold.

**Lemma 6 .**

(1) *A*(*K*) ⊂ *K* and *A* : *X* → *X* is a completely continuous operator.

(2) If there exists *ε* > 0 such that *f*(*x*(*t*)) ≤ *εx*(*t*) for *x* ∈ *K*, *t* ∈ [0, *ω*], then

(40) $$\begin{array}{c} \left\|\;Ax\;\right\|\leq \frac{3\varepsilon \sigma_{\infty }\omega }{1-\kappa }\left\|\;x\;\right\|. \end{array}$$

**Proof**

(1) By direct computation, for *x* ∈ *K* ⊂ *X*,

(41) Axt+ω=3σ∞∫t+ωt+2ωGt,sfxs−τds=3σ∞∫tt+ωGt+ω,s+ωfxs+ω−τds=3σ∞∫tt+ωGt,sfxs−τds=Axt.

Moreover, for *x* ∈ *K* ⊂ *X* and *t* ∈ [0, *ω*], one can get

(42) Axt3σ∞∫tt+ωGt,sfxs−τds≥3κσ∞1−κ∫0ωfxs−τds,Axt3σ∞∫tt+ωGt,sfxs−τds≤3σ∞1−κ∫tt+ωfxs−τds=3σ∞1−κ∫0ωfxs−τds.

Subsequently,

(43) Axt3σ∞1−κ∫tt+ωfxs−τds=3σ∞1−κ∫0ωfxs−τds.

Therefore, we can get

(44) $$\begin{array}{c} \left(\;Ax\;\right)\left(\;t\;\right)\geq \frac{\kappa }{1-\kappa }\left(\;1-\kappa \;\right)\left\|\;Ax\;\right\|=\kappa \left\|\;Ax\;\right\|. \end{array}$$

Hence *A*(*K*) ⊂ *K*.

Next, we prove that *A* : *X* → *X* is a compact operator. Since

(45) Axt3σ∞∫tt+ωGt,sfxs−τds≤3σ∞1−κ∫tt+ωfxs−τds=3σ∞1−κ∫0ωfxs−τds=3σ∞1−κ∫0ωxs−τpxs−τ3ds≤σ∞1−κ∫0ωxs−τds≤ωσ∞1−κx,

where we have used the fact 0 < *p*(*y*) < 1/3, *y* > 0, then we can get that *A* is uniformly bounded. Let *F*(*t*) = ∫_*t*_^*t*+*ω*^ *G*(*t*, *s*)*f*(*x*(*s* − *τ*)). One can show that

(46) Axt1−Axt2=3σ∞∫t1t1+ωGt1,sfxs−τds−3σ∞∫t2t2+ωGt2,sfxs−τds=3ωσ∞Ft1−Ft2=3ωσ∞F′ξ·t1−t2,

where the mean value theorem has been used and

(47) 0≤F′ξ=Gt,t+ωfxt+ω−τ−Gt,tfxt−τ+∫tt+ωGt′t,sfxs−τdst=ξ≤Gt,t+ωfxt+ω−τ+∫tt+ωGt′t,sfxs−τdst=ξ.

Then

(48) F′ξ131−κx+γ∗ω31−κx=1+γ∗ω31−κx.

It follows that

(49) Axt1−Axt2≤1+γ∗ωωσ∞1−κx·t1−t2.

Thus *A* is equicontinuous. By Arzela-Ascoli theorem, it follows that *A* : *X* → *X* is a compact operator. Therefore, it is a completely continuous operator.

(2) If there exists *ε* > 0 such that *f*(*x*(*t*)) ≤ *εx*(*t*) for *x* ∈ *K*, *t* ∈ [0, *ω*], by definition of (*Ax*)(*t*), one can get

(50) Axt3σ∞∫tt+ωGt,sfxs−τds≤3σ∞1−κ∫0ωfxs−τds≤3εσ∞1−κ∫0ωxs−τds≤3εσ∞ω1−κx.

This completes the proof.

**Lemma 7 (see [21–23]).**

Let *E* be a Banach space and *K* is a cone in *E*. For *r* > 0, *Ω*_*r*_ = {*x* ∈ *K* : ‖*x*‖ < *r*}. Assume that $A:{\overset{-}{\Omega }}_{r}\to K$ is completely continuous operator such that *Au* ≠ *u* for *u* ∈ ∂*Ω*_*r*_ = {*x* ∈ *K* : ‖*x*‖ = *r*}. Then we have the following:

(I) If ‖*Au*‖ ≥ *u* for *u* ∈ ∂*Ω*_*r*_, then *i*(*A*, *Ω*_*r*_, *K*) = 0.
(II) If ‖*Au*‖ ≤ *u* for *u* ∈ ∂*Ω*_*r*_, then *i*(*A*, *Ω*_*r*_, *K*) = 1.

*i*(*A*, *Ω*_*r*_, *K*) is the fixed point index of *A* on *Ω*_*r*_ with respect to *K*.

**Theorem 8 .**

Equation (11) has at least one positive *ω*-periodic solution for $\lambda^{*}<\sigma_{\infty }-\tilde{\sigma }$.

**Proof**

From definition of operator *A*, we can see that (11) admits oscillatory solutions whose period matches that of *λ*(*t*) (i.e., whose period is *ω*) if and only if *A* has fixed points.

For *r* > 0, define *Ω*_*r*_ = {*x* ∈ *K* : ‖*x*‖ < *r*} and ∂*Ω*_*r*_ = {*x* ∈ *K* : ‖*x*‖ = *r*}. If *x* ∈ ∂*Ω*_*r*_, let

(51) $$\begin{array}{c} \alpha =\sigma_{\infty }\underset{t\in \left[0,\omega \right]}{\min}\overset{t+\omega }{\underset{t}{\int }}G\left(\;t,s\;\right)ds. \end{array}$$

Since $\lambda^{*}<\sigma_{\infty }-\tilde{\sigma }$, it follows that

(52) $$\begin{array}{c} \alpha >\underset{t\in \left[0,\omega \right]}{min}\overset{t+\omega }{\underset{t}{\int }}G\left(\;t,s\;\right)\gamma \left(\;s\;\right)ds=1. \end{array}$$

By the fact ${lim}_{u\to 0+}\left(f(u)/u\right)={lim}_{u\to 0+}p(\sqrt[{3}]{u})=1/3$, we have that there exists a positive constant *r*_1_ such that

(53) $$\begin{array}{c} 3f\left(\;u\;\right)\geq \frac{u}{\alpha } \end{array}$$

when 0 < *u* ≤ *r*_1_. For *x* ∈ *K* with ‖*x*‖ = *r*_1_, by definition of operator *A*, we have

(54) Axt3σ∞∫tt+ωGt,sfxs−τds≥rσ∞α∫tt+ωGt,sds=rσ∞α∫0ωGt,sds≥r1=x.

Since

(55) limu→+∞⁡fuu=limu→+∞⁡pu3=0,limu→0+⁡fuu=limu→0+⁡pu3=13,

there exists 0 < *r*_1_ < *r*_2_ such that *f*(*u*) ≤ *εu* for 0 < *u* ≤ *r*_2_. Thus *f*(*x*) ≤ *εx* for *x* ∈ ∂*Ω*_*r*_2__ and *t* ∈ [0, *ω*]. By Lemma 6(2), we have

(56) $$\begin{array}{c} \left\|\;Ax\;\right\|\leq \frac{3\varepsilon \sigma_{\infty }\omega }{1-\kappa }\left\|\;x\;\right\|<\left\|\;x\;\right\|. \end{array}$$

It follows from Lemma 7 that

(57) iA,Ωr1,K=0,iA,Ωr2,K=1.

Thus it follows from additivity of the fixed point index that

(58) $$\begin{array}{c} i\left(\;A,\Omega_{r_{2}}\setminus {\overset{-}{\Omega }}_{r_{1}},K\;\right)=1. \end{array}$$

Therefore, *A* has at least one fixed point in $\Omega_{r_{2}}\setminus {\overset{-}{\Omega }}_{r_{1}}$ which is a positive *ω*-periodic solution to (11) for $\lambda^{*}<\sigma_{\infty }-\tilde{\sigma }$. This completes the proof.

## 3. Computer Simulations and Conclusions

In this paper a mathematical model for a solid avascular tumor growth under the effect periodic therapy with time delays in proliferation is studied. The periodic therapy can be interpreted as a treatment and *λ*(*t*) describes the rate of cell apoptosis caused by the periodic therapy. We mainly study how the periodic therapy influences the growth of tumors. We have derived a sufficient condition for the global stability of tumor free equilibrium and proved the existence of a periodic solution under some conditions. We also prove that if external concentration of nutrients is large the tumor will not disappear and the condition under which there exist periodic solutions to the model is determined. Hence, in biology sense, the results have practical significance in terms of determining the amount of drug required to eliminate the tumor and tell us that the tumor may grow in a periodic way under some conditions.

In [11, 19], the authors have studied the special cases of the model. In [11], the authors consider the case when *λ*(*t*) ≡ 0, and the results show that the tumor radius will tend to zero or tend to a stationary version. In [19], the author considers the case when *τ* = 0 and the result shows that the tumor radius will tend to zero or tend to a periodic version. From the analysis, we can see that the periodic therapy makes the tumor growth more complicated.

**Figure 2:**
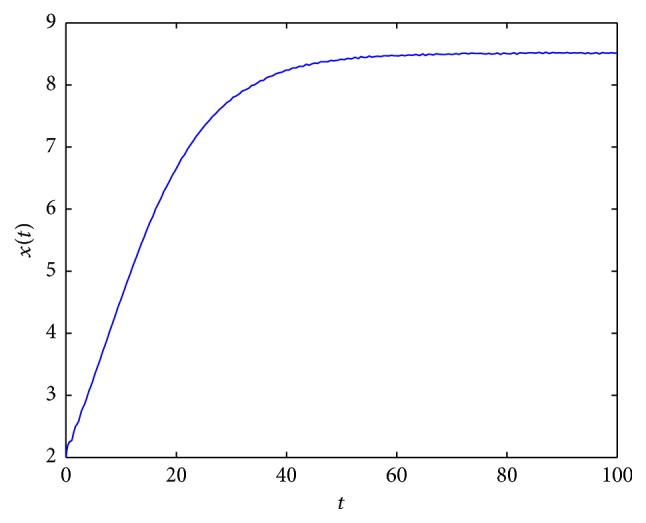
An example of solution to (11) for *σ*_*∞*_ = 5, *γ*(*t*) = 4, *τ* = 1, and *x*_0_ = 2.

**Figure 3 fig3:**
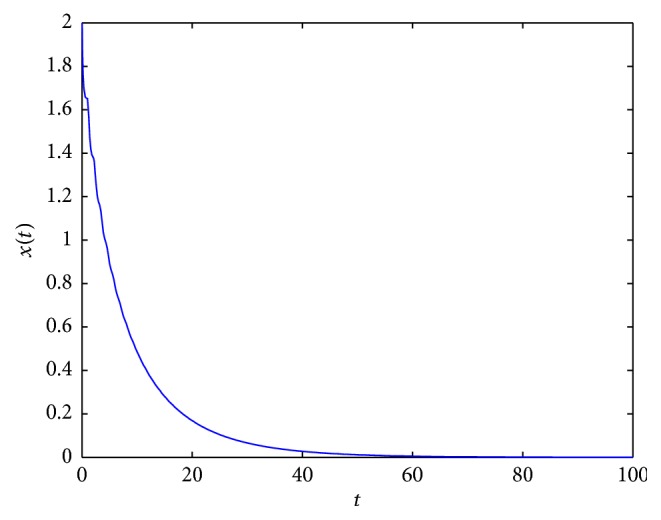
An example of solution to (11) for *σ*_*∞*_ = 5, *γ*(*t*) = 5.5, *τ* = 1, and *x*_0_ = 2.

**Figure 4 fig4:**
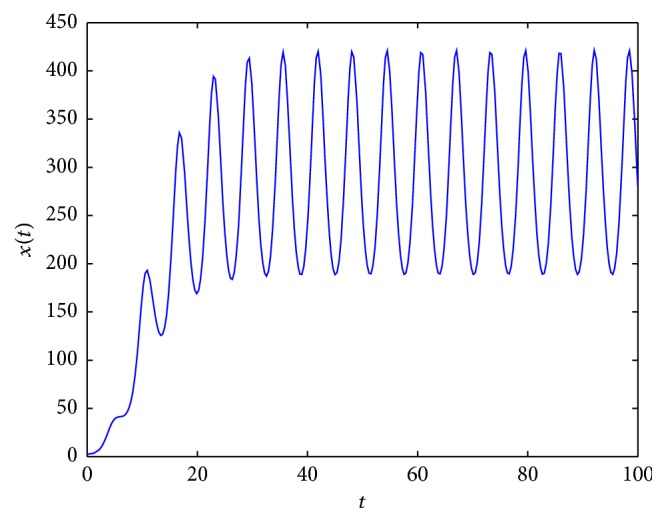
An example of solution to (11) for *σ*_*∞*_ = 5, $\tilde{\sigma }=1$, *γ*(*t*) = 2 + cos⁡(*t*), *τ* = 1, and *x*_0_ = 2.

**Figure 5 fig5:**
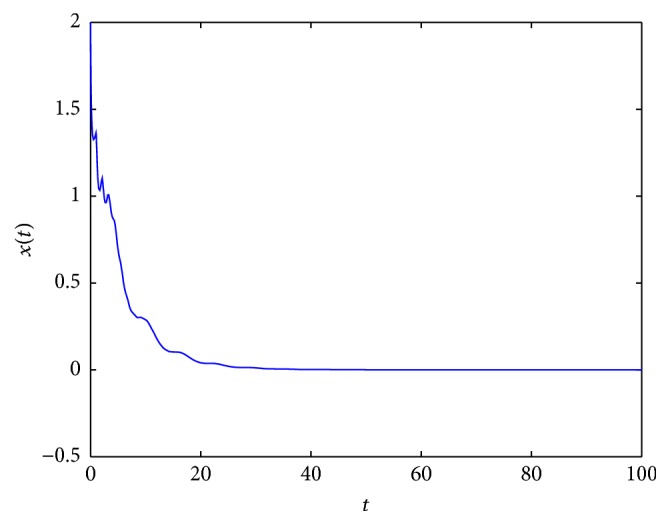
An example of solution to (11) for *σ*_*∞*_ = 5, $\tilde{\sigma }=1$, *γ*(*t*) = 6 + cos⁡(*t*), *τ* = 1, and *x*_0_ = 2.

**Figure 6 fig6:**
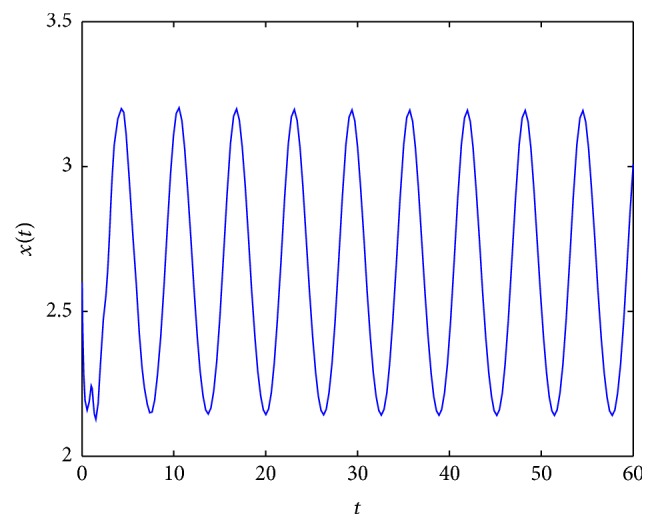
An example of solution to (11) for *σ*_*∞*_ = 5, $\tilde{\sigma }=3$, *λ*(*t*) = 1.5 + cos⁡(*t*), *τ* = 1, and *x*_0_ = 2.6.

**Figure 7 fig7:**
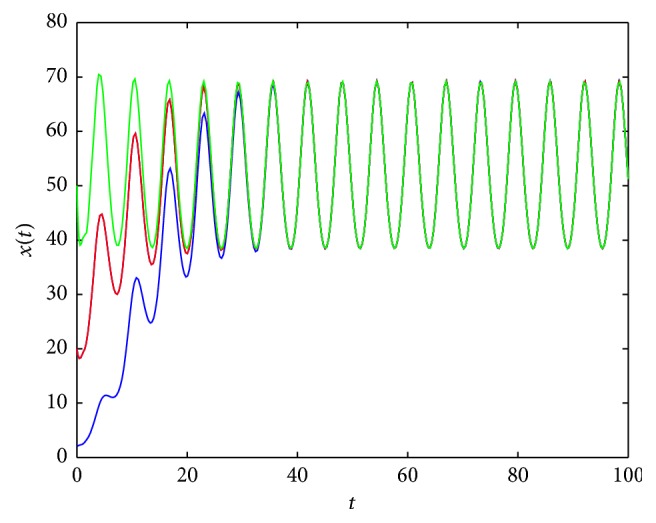
An example of solution to (11) for *σ*_*∞*_ = 5, $\tilde{\sigma }=1.5$, *λ*(*t*) = 1.5 + cos⁡(*t*), *τ* = 1, and *x*_0_ = 2,20,50, respectively.

**Figure 8:**
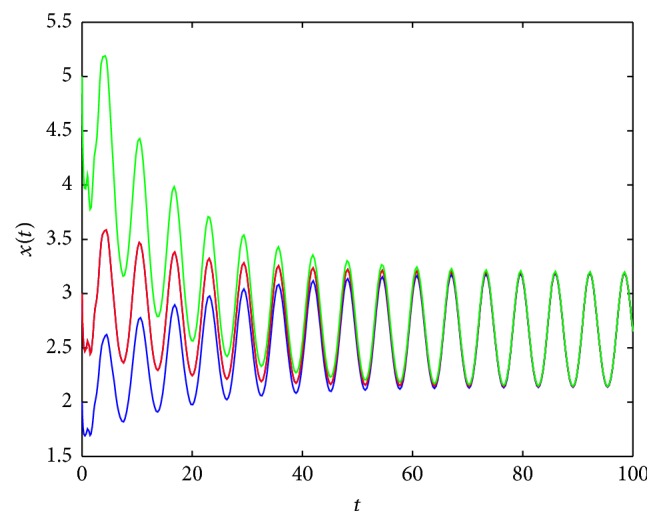
An example of solution to (11) for *σ*_*∞*_ = 5, $\tilde{\sigma }=3$, *λ*(*t*) = 1.5 + cos⁡(*t*), *τ* = 1, and *x*_0_ = 2,3, 5, respectively.

By using Matlab 7.1, we present some examples of solutions of (11) for different parameter values (see Figures 2 [Fig fig3] [Fig fig4] [Fig fig5] [Fig fig6] [Fig fig7]–8). For all simulations, the values used in simulations are given with the figures' captions.

In Figure 2, an example of the behaviour of solutions in the case which is covered by Lemma 3(2) is presented. In Figures 3 and 5, it occurs that, for various values of parameters, the tumor will disappear. First (see Figure 3), an example of the behaviour of solutions in the case which is covered by Lemma 3(1) is presented. And then (see Figure 5), an example of the behaviour of solutions in the case which is covered by Theorem 4(ii) is presented. In Figure 4, an example of the behaviour of solutions in the case which is covered by Theorem 4(i) and Theorem 8 is given.

From Figure 6, we see that when $0.5=\lambda_{*}<\sigma_{\infty }-\tilde{\sigma }=2<\lambda^{*}=2.5$, there exists a periodic solution. We conjecture that if $\overset{-}{\lambda }<\sigma_{\infty }-\tilde{\sigma }$, there exists at least one periodic solution to (11).

By Figures 7 and 8, we conjecture that the periodic solution is unique (if it exists) since, under different initial values, the solutions to (11) all tend to the periodic solution.

As being pointed out by a referee and we fully agree, the numerical simulations have limitations. Since we have no clinical data to compare with our results, the weaknesses of the numerical simulations are that we cannot use realistic parameter values to check whether the conditions for the existence of periodic solutions are biologically feasible and investigate (numerically) their amplitude. Further study on questions pointed out above and how we interpret them is needed.
